# Association between Information and Communication Technology Usage and the Quality of Sleep among School-Aged Children during a School Week

**DOI:** 10.1155/2014/315808

**Published:** 2014-01-28

**Authors:** Sandra Ononogbu, Marjut Wallenius, Raija-Leena Punamäki, Lea Saarni, Harri Lindholm, Clas-Håkan Nygård

**Affiliations:** ^1^School of Health Sciences, University of Tampere, Medisiinarinkatu 3, 33014 Tampere, Finland; ^2^School of Social Sciences and Humanities/Psychology, University of Tampere, Kalevankatu 5, 33014 Tampere, Finland; ^3^Tampere University of Applied Sciences, Kuntokatu 3, 33520 Tampere, Finland; ^4^Centre of Excellence of Health and Work Ability, Finnish Institute of Occupational Health, Topeliuksenkatu 41 aA, 00250 Helsinki, Finland

## Abstract

*Objective*. To determine the association between intensity of information and communication technology (ICT) usage and quality of sleep in school-aged children during a school week. *Methods*. In all 61 subjects, 10–14 years of age, a quasiexperimental laboratory study where criterions for inclusion were absence of prior medical condition and duration of ICT use. A portable device (Holter monitor) was used to measure heart rate variability (HRV) over a 24-hour period, while activity diary was used to record in 15-minute intervals ICT use and sleep and wake up time. Low and high ICT user groups were formed according to their intensity of ICT use. Statistical analysis was done with two independent samples tests and factorial ANCOVA. *Results*. The higher ICT users showed a lower sleep time standard deviation of normal to normal interval (SDNN) measures in comparison to the low ICT users. *Conclusion*. The intensive ICT use was associated with poorer quality of sleep indicated by physiological measures among children and adolescents. Knowing the crucial role of healthy sleep in this age, the results are reason for concern.

## 1. Introduction

The use of information and communication technology (ICT) such as computer use, internet surfing, and video game playing enhances certain academic skills [1]. However, the increase of intense ICT usage among adolescents [2] is associated with adverse effects such as poor psychosocial health status [3]. In addition, a reduction in average sleep duration due to delayed bedtime, early waking up, sleep disruption by nightmares, and sleep walking are related to the use of ICT during the night time [4]. The compromise of the restorative potential of sleep by a reduction in its quantity and quality is found to undermine the daytime functioning of adolescents, manifested as irritability, day time sleepiness, and inability to concentrate or assimilate during academic activities [5]. Long-term adverse health effects, like poor sleep, are related to depression [6]. Sleeping patterns of depressed subjects are shown to be irregular compared with the nondepressed subjects [7]. In addition, the probability of developing anxiety or depression is much higher among adolescents with poor sleep [6]. Also an increase of body mass index [8], abnormal glucose metabolism [9], predisposition to certain cardiovascular diseases such as hypertension [10], reduction of immune responsiveness to certain infection [11], increased risk of accidents [12], and incidence of substance abuse [13], aggravation of certain illnesses such as seizure disorders [14], and psychiatric symptoms [15] are associated with poor sleep among adolescents. Violent gaming may induce different autonomic responses in boys compared to nonviolent gaming—during playing and during the following night—suggesting different emotional responses [16].

Cardiovascular parameters such as heart rate, R-R interval, and blood pressure vary with sleep quality [17] and changes of heart rate variability (HRV) reflect the rhythm of sleep [18]. Under normal physiological conditions, sleep period is dominated by parasympathetic autonomic activity, which potentiates adequate recovery from the daytime stress. However, a severe stress disrupts recovery by raising sympathetic activity during sleep and HRV can be used as an indicator of sleep quality [19] The square root of the mean squared difference of successive normal to normal R-R intervals in time domain analysis of HRV in ECG (rMSSD) correlates to the high frequency bands (HF, 0.15–0.40 Hz) [20] and reflects the parasympathetic activity, which increases during sleep. On the other hand, the low frequency band (LF, 0.04–0.15 Hz) is related to heart rate and blood pressure, which increase with sympathetic dominance during daytime [21]. Those variations could be influenced by age, sex, life style and health status [22].

The aim of this study was to investigate whether and how the intensity of ICT usage among school-aged children is associated with the quality of sleep during a school week, indicated by blood pressure and cardiac autonomic control assessed by HRV.

## 2. Methods

### 2.1. Subjects

The study design is a quasiexperimental laboratory study. Participants were selected from those 222 (123 girls) fourth and 256 (137 girls) seventh graders from seven schools in the Tampere region of Finland who had earlier completed a survey questionnaire. The age groups were chosen according to the developmental saliency in transition from middle childhood to adolescence [23]. In the survey questionnaire participants had described their ICT use (mobile phones, computer games, internet surfing, and communication) including frequency (graded from 0 = never, 1 = less than once a week, 2 = 1-2 days a week, 3 = 3–5 times a week to 4 = almost daily) and daily duration (graded from 0 = not at all, 1 = less than an hour, 2 = 1-2 hours, 3 = 3-4 hours to 4 = over 4 hours), both during school days and the weekends. A subgroup of 88 pupils was derived from the survey questionnaire sample to attend the laboratory study using purposive sampling method. Subjects were stratified into fourth and seventh graders (10- and 13-year-olds) and boys and girls. To maximize the differences with respect to ICT use participants representing both low ICT use (never or 1 to 2 days a week and less than 1 hour at any time) and high ICT use (three to four hours or more, almost daily) were selected on the basis of the questionnaire data. Altogether 74 students participated in this study. The dropouts were due to refusals and unwillingness to provide informed consent on the part of either the schoolchildren or their parents.

### 2.2. Procedure

Before data collection the approval of the Ethical Committee of Pirkanmaa Hospital District (Code number R04050) was acquired. Permission was also obtained from the school principals. At each school an information meeting was held, usually for each participating class separately, and an information letter was delivered both for the pupils selected for the study and their parents. Written consents for the participation were obtained from all children willing to participate and their parent/guardian. The researcher gave directions for an activity diary and the subjects had possibility to ask questions.

At each school, in a peaceful room, the participants were given the Holter monitor and activity diary with verbal and written instructions. Next day children returned the Holter monitor and the activity diary to the researcher. Data were collected during three weeks, from the end of April to the beginning of May. The sampling day was always an ordinary school day. As a reward for taking part in the study, the subjects received a cinema ticket each.

### 2.3. Measures

#### 2.3.1. Intensity of ICT Use

Exposure to ICT was measured by use of a 24-hour activity diary during Holter's recordings. High subject compliance rate and data reliability have been obtained with diary method using objective measures as criterion, both among children and adolescents [24]. The participants were instructed to record in 15-minute intervals the time spent on different ICT activities with alternatives: (a) using mobile phone, for example, for phone calls or text messages, (b) playing mobile phone games, (c) playing TV or console games, (d) playing computer or Internet games, (e) using computer for homework, writing, and so forth, (f) using computer for communication, and (g) general surfing on the Internet. Participants also recorded their sleeping hours, from going to bed until waking up the next morning. Total time spent on different ICT activities was calculated summing up the 15 min intervals for each activity.

#### 2.3.2. Physiological Measures

Participants were required to have the monitor for a 24-hour duration and asked to record their bedtime and wake up time. ECG recordings were performed by three-channel ECG recorder during one workday and sampling rate was 128 Hz (Braemar DL700 Holter Monitor, Burnsville, USA). All recordings were first scanned by an experienced nurse specialized in the analyses of Holter's recordings. Thereafter every recording was rescanned by a physician and medical specialist in clinical physiology (HL). The HRV parameters were calculated using a validated software for long term ECG recordings (Century 2000, BMS Inc., St. Louis, USA). The standard time domain measures were obtained: standard deviation of all normal sinus R-R interval (SDNN, ms), root mean square of the successive normal sinus R-R interval difference (rMSSD, ms), percentage of successive normal sinus R-R interval longer than 50 ms (pNN50), and the heart rate computed from the mean cycle length of R-R complexes. The time domain parameters were used in further evaluation, because they are less sensitive to scanning errors and are preferred to be used in hourly analyses of long-term recordings instead of spectral analyses [24]. The body mass index (BMI) was calculated from the participant's reported height and weight using weight in kilograms/height in meters² formula. The mean systolic (MSBP) and mean diastolic (MDBP) blood pressure was measured. Prior to commencement of the study, participants were clinically checked by a specialized nurse and by an experienced researcher of the autonomic nervous system to exclude any cardiovascular or other chronic disorders that could confound heart rate variability recording.

### 2.4. Statistical Analysis

To answer the research questions, the participants were divided into two groups according to the intensity of ICT usage, that is, the total sum of hours spent in different ICT activities during Holter's recording. Six participants who had missing data on some sleep hours and additional seven participants with Holter's recording errors were excluded from the analysis. In the final sample there were 61 participants, with 27 (44%) high ICT users. In the high user group, the total time of ICT usage of 16 participants (62%) was one to less than 3 hours, of six participants (21%) 3 to less than 6 hours, and of five participants (17%) 6–8 hours. The rest of the participants (*N* = 34) were called low ICT user group who had not used ICT at all (72%) or had used ICT only for less than one hour.

Differences by gender and age in quality of sleep were tested by chi squared tests. The relationship between the intensity of ICT use and quality of sleep was tested with independent samples *t*-tests among the groups of high and low ICT users. Further analysis of the effects of gender, age, and body mass index on the outcome variables was done with factorial ANCOVA. The data analysis was carried out with SPSS 15.0 for Windows.

## 3. Results

Of the participants 32 were females (53%). The number of the participants in the younger age group (10-11 years) was 31 (51%), while in the older group (13-14 years) the number was 30 (49%) (Table 1). Among the high ICT user group, 52% belonged to the older age group (13-14 years). Participants in the high ICT user group differed significantly from the low ICT user group both in the total time used ICT and in using different form of ICT except for mobile phone use.

**Figure 1:**
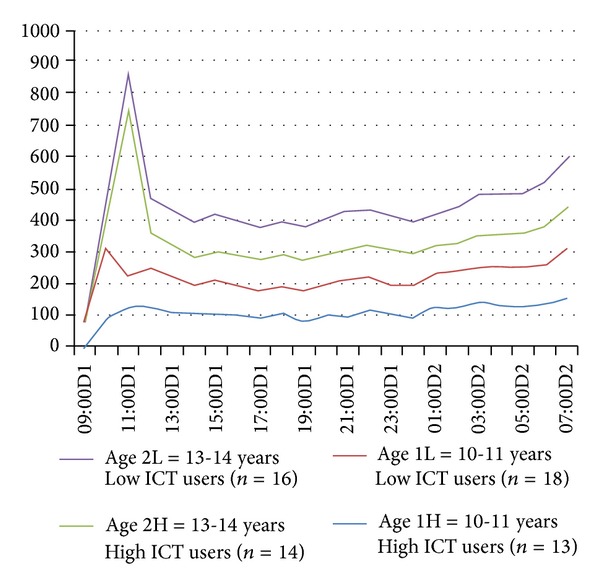
Mean HRV (SDNN, ms) during a school day and at night.

**Figure 2:**
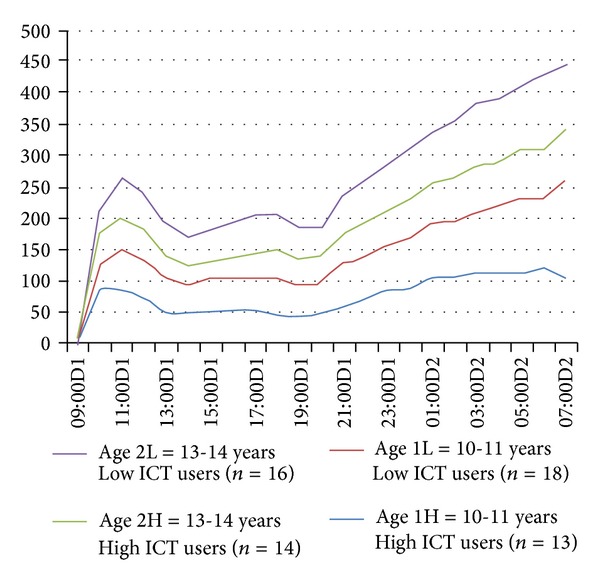
Mean HRV (rMSSD, ms) during a school day and at night.

The mean sleep time blood pressure did not differ between the ICT user groups. However, there was a statistically significant difference in HRV between the ICT user groups, when assessed with mean sleep time SDNN (*P* = 0.035) (Table 2). Comparison of the mean day and night HRV values between the ICT user groups revealed lower night values, especially in older (13-14 years) high ICT users. Nevertheless, analysis with rMSSD (*P* = 0.07) showed no statistically significant difference between the groups. HRV sleep period rose slower in high ICT users in both age groups compared to low ICT users (Figures 1 and 2). Adjustment for the influence of body mass index, gender, and age with factoral ANOVA resulted in no significant effect on the outcome (Table 3).

## 4. Discussion

This study revealed that HRV of high ICT users differed from low users during sleep on assessment with SDNN. High ICT users had lower level of night changes in heart rate variability. SDNN represents merely the overall cyclic variation of HRV and is correlated to diurnal oscillation of HRV. Low SDNN during the early night reflects the delayed readiness of the autonomic regulation for sleep. The result thus contributes to earlier findings showing that intensive ICT use has a negative impact on sleep pattern in adults [25]. Our earlier results revealed that long periods of ICT use were related to deteriorated stress regulation, indicated by flattened cortisol awakening response [26], which is in concordance with the current results. It can be also to suggest that among healthy children the intensity of vagal recovery is good after the sympathetic overdrive attenuating the cyclic component of HRV is blunted. However, similar to other studies [27], we did not find association between ICT and sleep quality when HRV is assessed with rMSSD. Taking account of the fact that rMSSD is reportedly a more sensitive indicator for parasympathetic activity [19], the influence of the small sample size on this outcome cannot be overruled.

Some mechanisms have been suggested to explain how or why ICT usage is associated with poor sleep. They include direct encroachment of sleep time by ICT use through its increased accessibility, affordability, addictive tendencies [28, 29], physiological changes induced by emotional reaction to activities [27], suppression of melatonin secretion [8] and reduction in physical activity [30]. Nevertheless, physical activity has also been shown to be insignificant in certain studies [30]. Further, ICT usage has been found to increase weight among adolescents as the displacement of physical activity with ICT usage is associated with a raise in body mass index [22]. In this study, we controlled the BMI in the analysis, and it was not associated with the quality of sleep, irrespective of gender and age.

The cross-sectional design of this study limits the ability to make causal inferences. It is difficult to rule out sleep disturbance as an actual precipitator of high ICT usage. The result of this study will need further evaluation with a prospective study with a randomized controlled design in which children are randomized to either a high ICT group or a control group, and also their sleep quantity and quality should be analyzed. In addition, potential confounders such as sleep environment, diet, parental socioeconomic status, physical activity, and other factors that could affect the state of the autonomic nervous system during sleep should be measured and adjusted for more effectively. Another limitation is the self-reports regarding intensity of ICT use, sleep duration, and wake up time, which warrants the reliability of the data. The accuracy of the data can be improved by employing a more comprehensive method in obtaining this information.

## 5. Conclusion

This study shows that the intensity of information and communication technology use by children and adolescents seems to interfere with the quality of sleep during a school week. The participants with low ICT use seem to sleep better than the high users. High amount of ICT use by children and adolescents may destroy good sleep pattern. In particular the sympathetic overdrive may continue to the early sleep and cause a delay in parasympathetic recovery. The length of restorative sleep might shorten, which is a health risk for physical and cognitive health among children. In experimental conditions prolonged video game playing caused disruption to adolescent sleep [31]. Because sleep is very important for adolescence growth, cognitive functioning, and physical health [32], the results are of concern. Further studies are needed with larger number of participants, comprehensive account of other forms of ICT use beyond computer use, internet surfing, and video game playing to enhance a more conclusive result of the effects of ICT use on children's and adolescents' sleep. The use of activity monitors would increase the possibility to assess the actual sleeping time. Research is also needed to explore whether the amount of ICT usage through interfering restorative sleep is related to adolescents' school performance (such as attention, persistence, or memory) or behaviour problems.

## Figures and Tables

**Table 1:** Characteristics of study participants.

Variables	*N* (%)	ICT use, *n* (%)			SDNN (ms)	rMSSD (ms)	BMI (kg/m^2^)	MSBP (mm/hg)	MDBP(mm/hg)
		Low users	High users	*P* value	Mean (SD)	Mean (SD)	Mean (SD)	Mean (SD)	Mean (SD)
Gender									
Males	29 (48)	19 (65)	10 (35)	0.143	108.35 (27.60)	80.45 (25.62)	17.72 (2.38)	114.81 (9.67)	75.45 (8.37)
Females	32 (52)	15 (47)	17 (53)		105.35 (28.10)	72.58 (27.14)	19.91 (3.25)	114.58 (7.87)	72.95 (6.31)
Age									
10-11 years	31 (51)	18 (58)	13 (42)	0.710	105.80 (26.41)	78.10 (26.77)	17.89 (2.79)	114.20 (8.45)	75.95 (7.45)
13-14 years	30 (49)	16 (53)	14 (47)		107.77 (29.40)	74.28 (26.57)	19.94 (3.03)	115.21 (9.04)	72.16 (6.90)

ICT: information and technology use; *P*: significant level; SDNN: Standard deviation of NN interval; rMSSD: root mean square of successive difference; BMI: body mass index; MSDP: mean systolic blood pressure; MDBP: mean diastolic blood pressure.

**Table 2:** Unadjusted means of SDNN (ms) and rMSSD (ms) by ICT use.

Variables	ICT use		Mean difference (95% CI)	*P* value
	Low users Mean (SD)	High users Mean (SD)
SDNN	114.12 (27.12)	99.05 (27.23)	15.07 (1.06–29.09)	**0.035**
rMSSD	82.61 (27.14)	69.87 (25.27)	12.73 (−0.85–26.32)	0.066

SDNN: Standard deviation of NN interval; rMSSD: root mean square of successive difference; ICT: information and technology use; *P*: significant level.

Bold means statistically significant.

**Table 3:** Means of SDNN (ms) and rMSSD (ms) by ICT use, adjusted for gender, age and BMI.

Variables	*N*	SDNN			rMSSD		
		Mean (SD)	*F* statistic	*P* value	Mean (SD)	*F* statistic	*P* value
ICT use							
Low users	34	114.12 (27.12)	11.17	**0.002**	82.61 (27.14)	7.84	**0.008**
High users	27	99.05 (27.23)			69.87 (25.27)		

SDNN: Standard deviation of NN interval; rMSSD: root mean square of successive difference; ICT: information and technology use; BMI: body mass index; *P*: significant level.

Bold means statistically significant.

## References

[1] Subrahmanyam K, Greenfield P, Kraut R, Gross E (2001). The impact of computer use on children’s and adolescents’ development. *Journal of Applied Developmental Psychology*.

[2] Statistics Finland (2006). *From Citizen to Ecitizen: Results from Statistical Surveys about Finns’ Use of ICT in 1996–2005*.

[3] Mathers M, Canterford L, Olds T, Hesketh K, Ridley K, Wake M (2009). Electronic media use and adolescent health and well-being: cross-sectional community study. *Academic Pediatrics*.

[4] Mesquita G, Reimão R (2007). Nightly use of computer by adolescents: its effect on quality of sleep. *Arquivos de Neuro-Psiquiatria*.

[5] Dahl RE (1996). The impact of inadequate sleep on children’s daytime cognitive function. *Seminars in Pediatric Neurology*.

[6] Johnson EO, Chilcoat HD, Breslau N (2000). Trouble sleeping and anxiety/depression in childhood. *Psychiatry Research*.

[7] Heins E, Seitz C, Schüz J (2007). Bedtime, television and computer habits of primary school children in Germany. *Gesundheitswesen*.

[8] Higuchi S, Motohashi Y, Liu Y, Ahara M, Kaneko Y (2003). Effects of VDT tasks with a bright display at night on melatonin, core temperature, heart rate, and sleepiness. *Journal of Applied Physiology*.

[9] Spiegel K, Leproult R, van Cauter E (1999). Impact of sleep debt on metabolic and endocrine function. *The Lancet*.

[10] Javaheri S, Storfer-Isser A, Rosen CL, Redline S (2008). Sleep quality and elevated blood pressure in adolescents. *Circulation*.

[11] Matsui Y, Saito K, Nakakuma T, Michi K (1991). Studies on the host factors in the outbreak of odontogenic infection—the background factors of patients and the effect of serum after sleep deprivation on PMN chemotaxis. *Kansenshogaku Zasshi*.

[12] Ohayon MM, Caulet M, Philip P, Guilleminault C, Priest RG (1997). How sleep and mental disorders are related to complaints of daytime sleepiness. *Archives of Internal Medicine*.

[13] Johnson EO, Breslau N (2001). Sleep problems and substance use in adolescence. *Drug and Alcohol Dependence*.

[14] Liamsuwan S, Grattan-Smith P, Fagan E, Bleasel A, Antony J (2000). The value of partial sleep deprivation as a routine measure in pediatric electroencephalography. *Journal of Child Neurology*.

[15] Paavonen EJ, Almqvist F, Tamminen T (2002). Poor sleep and psychiatric symptoms at school: an epidemiological study. *European Child and Adolescent Psychiatry*.

[16] Ivarsson M, Anderson M, Åkerstedt T, Lindblad F (2009). Playing a violent television game affects heart rate variability. *Acta Paediatrica*.

[17] Monti A, Medigue C, Nedelcoux H, Escourrou P (2002). Autonomic control of the cardiovascular system during sleep in normal subjects. *European Journal of Applied Physiology*.

[18] Zhuang Z, Gao X, Gao S (2005). The relationship of HRV to sleep EEG and sleep rhythm. *International Journal of Neuroscience*.

[19] Michels N, Clays E, de Buyzere M, Vanaelst B, de Henauw S, Sioen I (2013). Children's sleep and autonomic function: low sleep quality has an impact on heart tate variability. *Sleep*.

[20] Task Force (1996). Heart rate variability: standards of measurement, physiological interpretation, and clinical use. Task force of the European society of cardiology and the North American society of pacing and electrophysiology. *Circulation*.

[21] Carrington M, Walsh M, Stambas T, Kleiman J, Trinder J (2003). The influence of sleep onset on the diurnal variation in cardiac activity and cardiac control. *Journal of Sleep Research*.

[22] Singh RB, Cornélissen G, Weydahl A (2003). Circadian heart rate and blood pressure variability considered for research and patient care. *International Journal of Cardiology*.

[23] Steinberg L, Morris AS (2001). Adolescent development. *Annual Review of Psychology*.

[24] Lombardi F, Stein P (2011). Origin of heart rate variability and turbulence: an appraisal of autonomic cardiovascular function. *Frontiers in Physiology*.

[25] van Amelsvoort LGPM, Schouten EG, Maan AC, Swenne CA, Kok FJ (2000). Occupational determinants of heart rate variability. *International Archives of Occupational and Environmental Health*.

[26] Wallenius M, Hirvonen A, Lindholm H (2010). Salivary cortisol in relation to the use of information and communication technology (ICT) in school-aged children. *Psychology*.

[27] Garde AH, Laursen B, Jørgensen AH, Jensen BR (2002). Effects of mental and physical demands on heart rate variability during computer work. *European Journal of Applied Physiology*.

[28] Owens J, Maxim R, McGuinn M, Nobile C, Msall M, Alario A (1999). Television-viewing habits and sleep disturbance in school children. *Pediatrics*.

[29] Johnson JG, Cohen P, Kasen S, First MB, Brook JS (2004). Association between television viewing and sleep problems during adolescence and early adulthood. *Archives of Pediatrics and Adolescent Medicine*.

[30] Marshall SJ, Biddle SJH, Gorely T, Cameron N, Murdey I (2004). Relationships between media use, body fatness and physical activity in children and youth: a meta-analysis. *International Journal of Obesity*.

[31] King DL, Gradisar M, Drummond A (2013). The impact of prolonged violent video-gaming on adolescent sleep: an experimental study. *Journal of Sleep Research*.

[32] Gruber R (2013). Making room for sleep: the relevance of sleep to psychology and the rationale for development of preventative sleep education programs for children and adolescents in the community. *Canadian Psychology*.

